# The Antifreeze and Cryoprotective Activities of a Novel Antifreeze Peptide from *Ctenopharyngodon idella* Scales

**DOI:** 10.3390/foods11131830

**Published:** 2022-06-22

**Authors:** Meizhu Dang, Ruifeng Wang, Yangyang Jia, Jing Du, Ping Wang, Yawei Xu, Chunmei Li

**Affiliations:** 1 College of Food Science and Technology, Huazhong Agricultural University, Wuhan 430072, China; dangmeizhu@webmail.hzau.edu.cn (M.D.); wrf@webmail.hzau.edu.cn (R.W.); jiayangyang@webmail.hzau.edu.cn (Y.J.); zhanghailong@whpu.edu.cn (J.D.); xuyawei@webmail.hzau.edu.cn (Y.X.); 2 School of Energy and Intelligence Engineering, Henan University of Animal Husbandry and Economy, Zhengzhou 450002, China; 80423@hnuahe.edu.cn; 3 College of Food Science and Engineering, Wuhan Polytechnic University, Wuhan 430023, China

**Keywords:** antifreeze peptides, thermal hysteresis activity, scales, isolation, low-temperature protection

## Abstract

The purpose of this study is to obtain new antifreeze peptides (AFPs) that are natural, safe, and high activity from *Ctenopharyngodon idella* scales. The optimal hydrolysis conditions were investigated, and chromatography-based isolation was conducted using thermal hysteresis activity (THA) as an index. Molecular dynamic simulation (MDs) was explored to reveal the antifreeze mechanism of the AFPs. The results showed that the optimal hydrolysis conditions were 4000 U/g papain enzyme for 60 °C at pH 5.0 and substrate concentration (1:10) for 3 h, as unveiled by single-factor experiment results. The AFPs documented a THA of 2.7 °C when the T_h_ was 1.3 °C. Hydrophilic peptide, named GCFSC-AFPs, with a THA of 5.09 °C when the T_h_ was 1.1 °C was obtained after a series isolation of gel filtration, ion exchange, and reversed-phase HPLC chromatography. The AFPs had a molecular weight of 1107.54~1554.72 Da with three main peptides in the amino acid sequence of VGPAGPSGPSGPQ, RGSPGERGESGPAGPSG, and VGPAGPSGPSGPQG, respectively. The survival rate of yeast with GCFSC-AFPs reached 84.4% following one week of exposure at −20 °C. MDs indicated that GCFSC-AFPs interfered with the ice-water interaction and thus inhibited the ice crystallization process. Our data suggested that the GCFSC-AFPs were a novel and potential antifreeze agent in the food industry.

## 1. Introduction

With the development of modernization and industrialization, the low-temperature cold chain technique has become increasingly important for preserving foods. As the most commonly used technology, frozen storage is widely used in many foods. However, the food quality and taste are extremely reduced during freeze–thawing due to ice crystal growth and recrystallization. Although commercial antifreeze agents such as sucrose and sorbitol are used in food such as surimi products and other frozen foods, they contain high sugar and calories, which may bring not only undesired flavor and taste but also excess food energy. In addition, common biological systems used cryoprotectants such as ethylene glycol, glycerol, and dimethyl sulfoxide that are not fit for food products due to the concerns regarding toxicity and changes to the natural taste and texture of the food. Therefore, developing alternative antifreeze peptides (AFPs) is necessary.

Due to consumer needs for nutrition and health, foodborne antifreeze glycoproteins and polypeptides (AFPs) have attracted the increasing interest of food scientists worldwide. AFPs can reduce the freezing point of solutions, modify the morphology of ice crystals, inhibit recrystallization, and improve the survival rate of microorganisms in frozen storage. AFPs have been considered natural ice crystal growth inhibitors for use in frozen foods (such as dough, meat, fish, desserts, fruits, and vegetables) and have been studied widely [1,2]. As cryoprotectants or food ingredients, AFPs have numerous advantages with regard to safety, health, and nutrition. They have played an important role in food freezing, frozen storage, and cryogenic tissue engineering. They have become promising cryoprotectants and food ingredients with broad market demand and application prospects. In addition, compared with antifreeze proteins, antifreeze polypeptides have more stable physicochemical properties as well as better low-temperature protection and antifreeze activity. It was reported that polypeptide with repeat Gly-X-Y sequence (where X is mostly proline or hydroxyproline and Y is any other amino acid residue) and molecular weight of 1000 to 3000 Da usually exerted antifreeze potential. For example, two AFPs rich in glycine and Gly-X-X repeats were extracted from snow fleas by Graham and Davies [3]. There are also many reports on antifreeze peptides separated from pig skin [4], bovine bone [5], fish skin [6], and shark skin [7]. However, outbreaks of swine fever, foot-and-mouth disease, etc., brought safety anxiety related to using antifreeze proteins and antifreeze polypeptides extracted from mammals; this resulted in shifting the attention of antifreeze polypeptides from aquatic products.

Because collagen from different species varies significantly in its amino acid composition/sequence and hydroxyproline content, the structure of peptides obtained from different species of fish collagen might be different. Therefore, their antifreeze potential also might vary considerably. For example, Chen et al. [8] prepared active AFPs from tilapia scales using enzymatic hydrolysis and found that its thermal hysteresis activity (THA) was 0.29 °C. Sun, et al. [9] obtained SaAFP from salmon skin gelatin using enzymatic hydrolysis with THA of 0.53 °C. By contrast, the THA of AFPs from *Scomberomorus niphonius* skin was proved to be 1.7 °C by Fu et al. [10]. Cao et al. [11] isolated ice-binding collagen peptides with a THA as high as 5.28 °C. Past work has shown that the reason for the differences in the antifreeze ability of collagen antifreeze peptides from different species is closely related to the source.

Because the thermal denaturation temperature of fish collagen is lower than that of mammalian collagens [12] and fish collagen contains lower hydroxyproline content than mammalian collagens, physicochemical properties such as the ice crystal growth-inhibiting properties of peptides released from fish collagen/gelatin under similar enzymatic digestion conditions might be different from mammalian collagen/gelatin hydrolysate [6]. Similarly, the physicochemical properties of peptides released from freshwater fish under similar enzymatic digestion conditions might differ from ocean fish collagen/gelatin hydrolysate. However, currently, reports on AFPs with high THA and potential application from freshwater fish are few.

*Ctenopharyngodon idella*, commonly known as grass carp, is an essential freshwater cultured fish species in China. In 2018, grass carp generated over 5.5 million tons of freshwater food. Incomplete statistics suggested that about 100 kilotons of grass carp scales are produced annually (Ministry of Agriculture and Fisheries, 2018). These by-wastes are excellent raw materials for high-value-added products such as antifreeze peptides. However, reports on antifreeze polypeptides from fish scales are scarce. Therefore, this paper aimed to obtain highly active antifreeze peptides using grass carp scales as raw materials by controlling enzymatic hydrolysis. We believed that our work might help reveal new antifreeze peptides and realize the high utilization of fish scales by-products.

## 2. Materials and Methods

### 2.1. Materials and Chemicals

*Ctenopharyngodon idella* scales were purchased from a local supermarket (Wuhan, China), washed, dried, and stored at room temperature until use. Novozymes (Beijing, China) Investment Co., Ltd. was the source of the alcalase, papain, trypsin, neutral protease, and pepsin protease and bovine serum albumin (BSA). Sephadex G-50 and ion exchange (sulfopropyl-Sephadex C-25) were provided by Yuanye Chemical Scientific Co. (Shanghai, China). All chemicals employed were reagent grade or higher.

### 2.2. Pretreatment of Ctenopharyngodon idella Scales

*Ctenopharyngodon idella* scales were crushed using a high-speed pulverizer. The powder was mixed with deionized water at a substrate concentration. The mixture was stirred ultrasonically (SB-5200DT, Ningbo, China) for 50 min at 45 °C and then decalcified in 1 mol/L of the citric acid solution for 24 h. After cleaning, the mixture was hydrolyzed under the following conditions. 

### 2.3. Screening Proteases 

The amount of 5 g of pretreated *Ctenopharyngodon idella* scales was mixed with distilled water at a ratio of 1:20 (*w*/*v*), and the samples were hydrolyzed with five proteases (neutral protease, alkaline, papain, trypsin, and pepsin protease) under optimum enzymolysis conditions. The optimal conditions for the proteases were as follows: pH 9 and 50 °C for alcalase; pH 7 and 55 °C for papain; pH 8 and 50 °C for trypsin; pH 2 and 45 °C for pepsin; and pH 7 and 55 °C for papain. Microbial cryogenic protection was used as the index for comparison. THA was used as the index for further comparison.

### 2.4. Optimizing AFP Preparation Conditions

Single-factor tests were based on enzyme dosage, reaction temperature, substrate concentration, and pH. A preliminary study was performed before finding out the data range of the study and optimizing the experiment. Preliminary experiments were conducted to optimize the enzyme and reaction parameters: enzyme dosage (3000, 3500, 4000, and 4500 U/g), temperature (40, 45, 50, 60 °C), substrate concentration (1:20, 1:15, 1:10), and pH (3, 4, 5, 6). THA was used as the index for comparison.

### 2.5. Probing the Protection from Hypothermia

The approach of Jia et al. [13] was employed to gauge the in vitro antifreeze activity of the samples. First, 1 g of hair was put in a 50 mL centrifuge tube followed by the addition of active dry yeast (*w*/*w*) in distilled water to activate the hair yeast and placement on the shaking table (Jintan, China) at 10 °C for 20 min. Then, 0.5% of the yeast slurry was added to the experimental group and mixed in a vortex mixer (Vortex-1, Shanghai, China) to be frozen at −20 °C. The frozen yeast suspension was thawed at 4 °C for 2 h and placed on the shaking bed to score the activity. Post-thawing, the yeast suspension was diluted: 0.1 mL of yeast suspension was absorbed and coated on a YPD plate, incubated for two days at 28 °C at a constant temperature, and the survival rate of yeast was calculated (the survival rate of yeast before freezing was set at 100%).

As elucidated above, yeast suspensions for the test and blank groups were prepared, separated, and kept at −20 °C. The suspensions were frozen for 7 d. After the suspensions were taken out, they were cultured, and the survival rate of the yeast was calculated:

$$Survival\;rate\;\left(\%\right)=\frac{CFU\;of\;survival\;cells\;post-freezing}{CFU\;of\;original\;cells\;without\;freezing}\times 100$$

(1).

### 2.6. THA Determination

This entailed the use of differential scanning calorimetry (DSC) on a DSC200 F2 instrument (NETZCH, Selb, Germany) as elucidated earlier [14,15] with slight modifications. Naphthalene was employed for temperature and baseline corrections prior to all assays, while BSA was used as a standard for GCAFP-free activity. Samples of 5 μL (20 mg/mL) were placed on an aluminum pan (the reference was the empty pan). After the DSC instrument was filled with nitrogen, the sample was cooled from 20 to −20 °C at a rate of 5 °C/min. This −20 °C was maintained for 5 min, followed by sample heating at 1 °C/min for partial melting. This was held (T_h_, hold temperature) for 2 min followed by cooling to −25 °C. THA was defined as T_h_ − T_o_ with T_o_ as the crystallization temperature. This `scheme was repeated at several T_h_ values to gauge the T_o_ values.

The THA and ice crystal content (φ) were computed according to Equations (2) and (3):

THA = T_h_ − T_o_ (2)

(3) $$\varphi =(1-\frac{-\Delta \mathrm{Hr}}{\Delta \mathrm{Hm}})\times 100$$

ΔHr is the crystallization enthalpy, and ΔHm is the melting enthalpy (both in J/g).

### 2.7. Isolating the Ice-Binding Collagen Peptides

#### 2.7.1. Gel Filtration Chromatography

The hydrolysate was first fractionated using a Sephadex G-50 gel filtration column (2.6 cm diameter × 100 cm length) followed by elution (5 mL/tube) with PBS water at pH 7.0 at a flow rate of 0.8 mL/min. The absorbance at 225 nm was scored, followed by THA analyses. Fractions that demonstrated higher antifreeze activity were pooled, followed by lyophilization for the ensuing step.

#### 2.7.2. Isolation and Purification by Ion-Exchange Chromatography

The “best performers” were loaded in an SP-Sephadex C-25 strong cation exchange column (1.6 cm diameter × 20 cm length) and equilibrated with 20 mM HAc-NaAc buffer (pH 5.0), followed by resolution employing 0–1.4 M NaCl in this buffer at 0.5 mL/min and probing of the fractions at 225 nm. The antifreeze activity of the pooled unadsorbed and adsorbed portions was scrutinized with the pooling and lyophilization of those that demonstrated high activity.

#### 2.7.3. Reversed-Phase HPLC

After ion-exchange chromatography, fractions with the highest THA activity were loaded onto a C18 column (10 mm diameter × 250 mm length; room temperature; flow rate: 1 mL/min and 220 nm detector). Following column equilibration employing 4% acetonitrile for 4 min post-sample injection, elution ensued for 15 min with a linear gradient of 4–100% acetonitrile solution and 100% acetonitrile solution for 5 min. Those that demonstrated significant absorbance were scored. The scrutiny of THA activity entailed the same conditions listed here (HPLC system: Agilent, CA, USA), with those fractions that demonstrated high absorbance scored.

### 2.8. Peptide Sequence Analysis by Q Exactive LC-MS/MS

This entailed employing an Ultimate 3000 HPLC-MS/MS system (Q-Exactive, Thermo Scientific) to probe the fractions that demonstrated evident antifreeze activity. The scheme was elution from the column into the mass spectrometer, followed by peptide sequencing retrieval and comparison analyses. The raw mass spectrometry test files were retrieved by the Uniport database with Mascot software for retrieval and comparison. LC analysis entailed loading 1 µL of the sample in an Accucore 18-MS (2.1 mm, 100 mm, 2.76 μm) column in a UPLC system and PDA detector (Waters Acquity; 0.2 mL/min flow rate; 45 °C; 200–600 nm-UV-detection) with a linear gradient of 1–30% acetonitrile solution for 45 min for elution, 30–90% acetonitrile solution for further 5 min for elution, 90% acetonitrile solution for additional 2 min for elution, 90–1% acetonitrile solution for further 5 min for elution. MS analysis was performed using a Q Exactive QTF MS system (Thermo Fisher). The parameters for the analysis were as follows: reflector, positive; mass range, 50–2000 *m*/*z*; ESI source temperature, 100 °C; desolvation temperature, 250 °C; capillary voltage, 3.8 kV; photomultiplier tube voltage, 1600 V. Control was exerted at: scan range, 100 to 2000 *m*/*z*, full scan resolution, 70,000; source temperature, 100 °C; MS/MS scan resolution, 17,500.

### 2.9. Homology Modeling of AFPs

The 3D structure of AFPs can be constructed based on homology modeling [16]. First, sequences of AFP were put into the protein sequence database using BLAST [17] to search for the template protein. Then a template (PDB: 3HQV) was selected that exhibited the highest sequence identity. Next, sequences of AFP and the template proteins were loaded in the PEP-FOLD3 server, which is a faster denovo structure prediction for peptides [18].

After that, the best model constructed was evaluated through the Ramachandran plot [19], which is geometrical validation. Then we found that there was one outlier (phi, psi) at peptide 2 (11 Gly) and peptide 3 (6 Pro), respectively, meaning that these amino acids were in disfavored conformations. Subsequently, the 3D structure of the peptides was optimized by molecular dynamic simulation using the GROMACS 5.1.4 software (University of Groningen, (Groningen, The Netherlands))package [20,21]. Briefly, an OPLS-AA all atom force field [22] was used to construct the topological file of the peptide, and the linear constraint solver (LINCS) algorithm [22] was applied to constrain all bonds. A cutoff of 1.0 nm was used for the coulombic and electrostatic interactions along with the particle mesh ewald (PME) method [23]. V-rescale and Parrinello-Rahamn methods were used to maintain the temperature and pressure, respectively. The optimized model was evaluated once again through a Ramachandran plot, and there were no outliers in the optimized model. 

### 2.10. Molecular Dynamic Simulation of Ice-Water-Peptide System

First, the ice model was obtained from www.ks.uiuc.edu and is shown in Figure 7. Then, the peptide was placed in the center of the box and solvated with water molecules. The initialized system is shown in Figure 7. After that, the system was minimized using the steepest descent algorithm, and the periodic boundary conditions were used for all systems. Prior to the production simulations, the systems were equilibrated with the V-rescale temperature thermostat and Parrinello-Rahamn pressure thermostat. The temperature was maintained at 260 K, and the pressure was maintained at 1 bar. Then, the production simulations were carried out at least 200 ns using the GROMACS 5.1.4 software package, and snapshots were saved every 2 ps. The simulation detail was analyzed through the built-in module, and snapshots were visualized using VMD 1.9.2 software(University of Illinois at Urbana-Champaign, (Urbana, IL, USA)) [24].

### 2.11. Data Processing and Statistical Analysis 

Statistical significance was determined by one-way analysis of variance with Dunnett’s post hoc test using SPSS 24.0 software (IBM SPSS for Windows, SPSS Inc., Chicago, IL, USA). Significance was set at *p* < 0.05. Data were expressed as the mean ± SD.

## 3. Results and Discussion

### 3.1. Optimizing the AFPs Preparation Conditions

**Figure 1:**
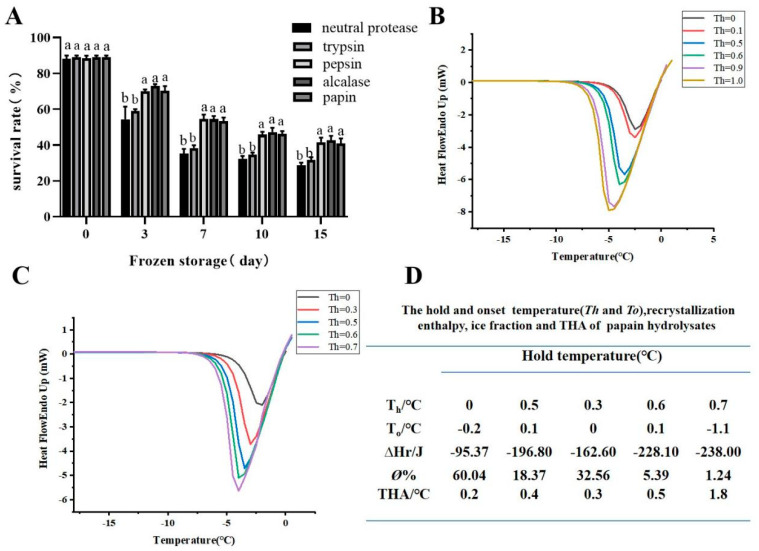
The hypothermia protective effects of 0.5% hydrolysates (trypsin, pepsin, alcalase, neutral protease, and papain) on yeast following 0, 3, 7, 10, and 15 d of freezing at −20 °C (**A**). The DSC curves (**B**) of *Ctenopharyngodon* *idella* scale hydrolysates after enzymolysis with alcalase. The DSC curves (**C**) and THA activity (**D**) of *Ctenopharyngodon* *idella* scale hydrolysates after enzymolysis with papain. The data points represent mean values calculated from two separate experiments. Note: Means with the same letter in the same column showed no significant difference (*p* ≥ 0.05).

The antifreeze activity of antifreeze proteins or peptides depends primarily on the domain of the local specific peptide chain, rather than the holoprotein [25,26]. Enzymatic hydrolysis is a common strategy used to liberate AFPs from bioresources [8,27]. Studies have found that the antifreeze activity of AFPs is closely related to the presence of specific amino acids, such as Gly, Ala, Thr, Asp, and Ser [2,6]. The enrichment of the above amino acids and Pro and hydroxyl groups in collagen peptides may be the key to improving their antifreeze activity. Therefore, alkaline protease, papain, trypsin, neutral protease, and pepsin were selected as the enzyme sources for hydrolyzing grass carp scales, and the effects of the different enzymes on the antifreezing effects of enzymatic hydrolysates under low temperature were studied. As shown in Figure 1A, peptides obtained with papain showed a yeast survival rate 53.43% after 7 d of frozen storage, which was significantly higher than those for trypsin (38.33%) (*p* < 0.05) and neutral protease (35.33%) (*p* < 0.05), but close to those for pepsin (52.10%) (*p* > 0.05) and alkaline (54.47%) (*p* > 0.05). Many previous studies showed that alkaline generally had better enzymatic hydrolysis effects. In order to accurately assess their antifreeze activity, the thermal hysteresis activity of peptides obtained with papain and alkaline was analyzed using DSC. As shown in Figure 1B–D, when the hold temperature (T_h_) was 0.8 °C, the THA of peptides obtained with papain was 1.8 °C (*p* < 0.05), which was higher than that of alkaline protease hydrolysate with THA of 0.9 °C at T_h_ of 1.0 °C. Our result was consistent with the research results in [9]. This difference might be related to the different enzyme digestion sites of protease. Papain is a thiol protease that cleases the carboxyl ends of arginine and lysine in proteins and peptides. The exposure to papain digestion was more conducive to antifreeze activity [9], and therefore, papain was chosen as the enzyme for the hydrolysis of the grass carp scales.

By controlling the cutting conditions of endonuclease protease, the active polypeptide with specific peptide chain length and structure composition could be produced, and the peptide with potent antifreeze activity could thereby be obtained efficiently. Therefore, we optimized other conditions, including the enzyme dosage, enzymolysis temperature, pH, and substrate concentration. Thermal hysteresis is defined as the difference between the melting point and the nonequilibrium freezing point of a solution, and it is used as an indicator of AFPs activity. At present, THA is commonly measured by the direct microscopic observation of crystal growth [14]. In this study, to obtain hyperactive antifreeze collagen peptides and accurately assess their antifreeze activity, we used the DSC method, which is simple, sensitive, and stable for determining the THA activity of collagen peptides [28].

**Figure 2:**
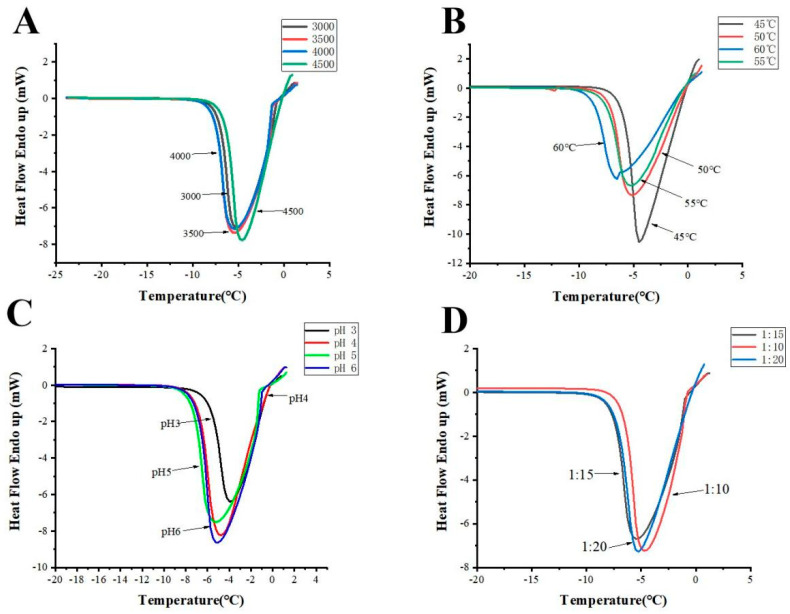
DSC curves (**A**–**D**) of *Ctenopharyngodon idella* scale hydrolysates after enzymolysis with different concentrations of papain (**A**), hydrolysis temperatures (**B**), pH (**C**), and substrate concentrations (**D**).

As shown in Figure 2A and Table 1, except for substrate concentration, the other three factors significantly impacted the THA (*p* < 0.05). The THA of hydrolysate with papain concentration of 4000 U/g was 2.70 °C, which was higher than other concentrations. However, a further increase in papain concentration from 4000 U/g to 4500 U/g caused an evident lowering of THA (0.80 °C) (*p* < 0.05).

The effect of temperature on THA was also measured. As shown in Figure 2B and Table 1, THA was the highest at 60 °C. The optimum temperature for papain is 55~65 °C.

The THA of the hydrolysates strongly depended on papain concentration and pH (Figure 2C and Table 1). The values reached the maximum (2.68 °C) when pH was 5.0, significantly higher than the values at pH 3.0 and 4.0. However, when pH was further increased to 6, the THA of the hydrolysate decreased to 2.0 °C.

Increasing the substrate level from 1:10 to 1:20 induced a minor alteration in THA (Figure 2D and Table 1). The THA of the hydrolysates decreased with the decrease in substrate concentration. When the solid–liquid ratio was 1:10, the THA of the enzymolysis solution reached the maximum (1.90 °C), similar to the results found by Jinhong and Pingfan [28]. When the solid–liquid ratio is lower than 1:10, the viscosity is higher. It is not conducive to enzymatic hydrolysis.

Taken together, the optimized conditions for AFPs preparation were as follows: papain level of 4000 U/g, temperature, 60 °C, solid–liquid ratio 1:10, pH 5.0, 3 h. Under the above optimum conditions, the THA of the hydrolysates could reach 2.7 °C.

### 3.2. Isolating Promising AFPs

**Figure 3:**
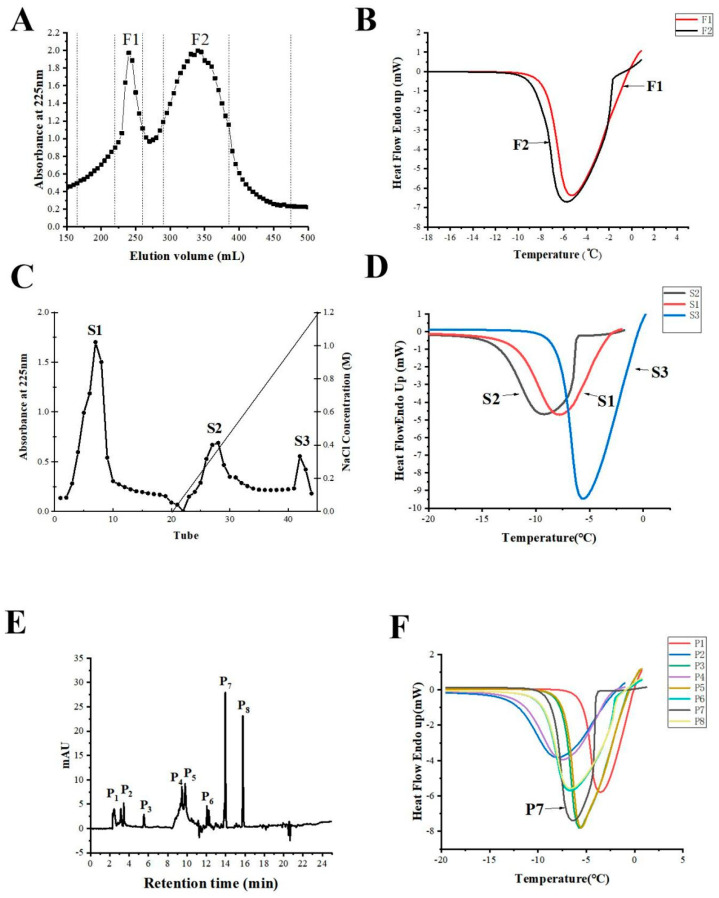
The elution and DSC curves of the AFPs. (**A**) The Sephadex G-50 gel elution profiles of the hydrolysates. (**B**) The corresponding DSC curves of the fractions (F1–F2). (**C**) The SP-Sephadex C-25 strong cation exchange F2 elution profiles. (**D**) The DSC curves of the fractions (S1–S3) eluted from the same column as (**C**). (**E**) The elution profile of the S2 fraction on a C18 column. (**F**) The DSC curves of fractions (P1, P2, P3, P4, P5, P6, P7, P8) eluted from C18 column. The data points represent the mean values calculated from two separate experiments.

The hydrolysates were first separated on a Sephadex G-50 gel filtration column. Two fractions (F1, F2) were obtained (Figure 3A). As shown in Figure 3B, compared with F1, F2 showed more effective antifreeze activity, as evidenced by a longer delay in the onset of refreezing for F2, with a maximal THA of 2.7 °C (Figure 3B, Table 2) (*p* < 0.05). In contrast, as the temperature fell, the re-crystallization of partially melted F1 was only delayed slightly. Therefore, the F2 fraction was pooled, lyophilized, and then loaded onto an SP-Sephadex C-25 strong cation exchange chromatographer for further fractioning. The elution profile is shown in Figure 3C. The figure shows that F2 was separated into three fractions, S1, S2, and S3, and that the THA of the adsorbed cationic peptides (S2), which mainly contained cationic peptides, was higher than the fraction (S1); additionally, the THA increased to 4.45 °C (Figure 3D, Table 2) (*p* < 0.05). The amount of the S3 fraction was minimal, so we did not collect it.

In order to obtain antifreeze peptides with higher THA, the S2 fraction was subsequently loaded onto a C18 reversed-phase high-performance liquid chromatography column, and the elution profile is shown in Figure 3E. Seven fractions were obtained, and the P7 fraction indicated the highest antifreeze activity with a THA of 5.09 °C (*p* < 0.05), significantly higher than the THA of the other fractions (Figure 3F, Table 2). The AFP purification folds from the *Ctenopharyngodon idella* scales are summarized in Table 2. After purification, the THA of the AFP increased 1.9-fold.

**Figure 4:**
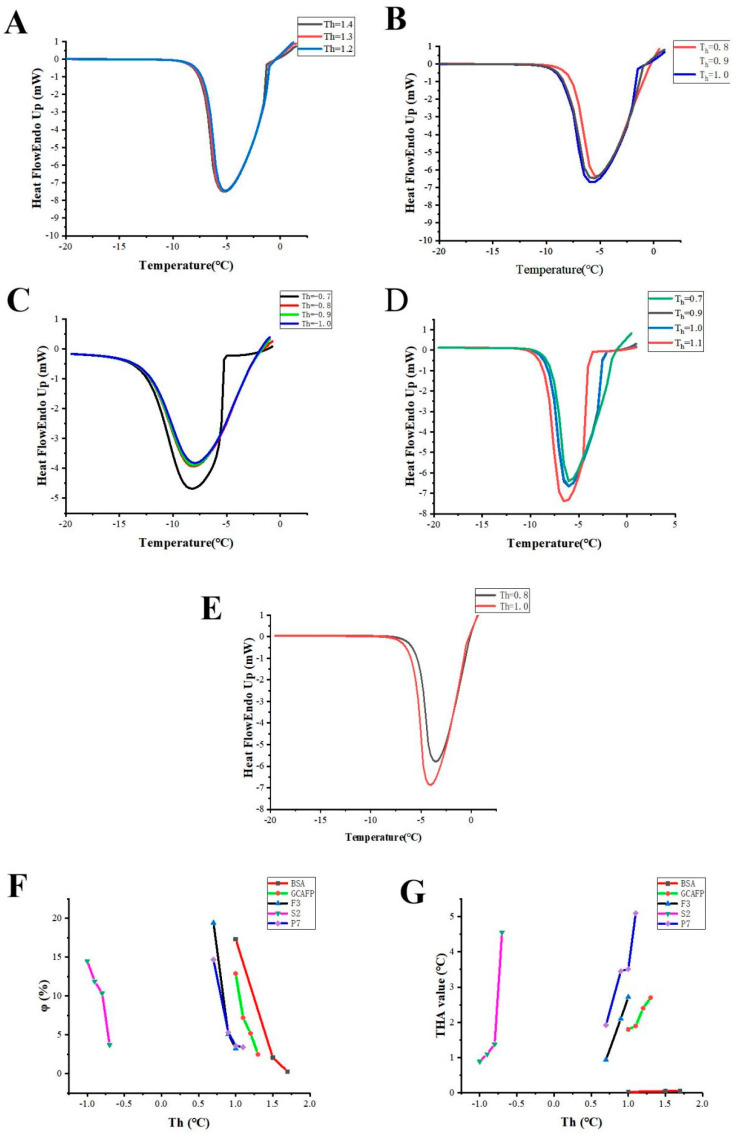
The thermal hysteresis activity (THA) of F2, S2, and P7 compared with GCAFP (original hydrolyzed *Ctenopharyngodon* *Idella* scales), as well as the negative control of BSA (AFP-free protein). The differential scanning calorimetry (DSC) typical curves of the freezing and melting processes at different hold temperatures (T_h_) for (**A**) GCAFP, (**B**) F2, (**C**) S2, (**D**) P7, and (**E**) BSA. The T_h_ values of the curves in (**A**) were 1.1, 1.2, and 1.3 °C, respectively. The T_h_ values of the curves in (**B**) were 0.8, 0.9, and 1.0 °C, respectively. The T_h_ values of the curves in (**C**) were −0.7, −0.8, −0.9, and −1.0 °C, respectively. The T_h_ values of the curves in (**D**) were 0.7, 0.9, 1.0, and 1.1 °C, respectively. The T_h_ values of the curves in (**E**) were 0.8 and 1.0 °C, respectively. The T_o_ value of each curve is in parentheses. Ice fraction-Th plots of BSA, GCAFP, F2, S2 and P7 (**F**). Data points represent mean values calculated from two separate experiments. THA-T_h_ plots of BSA, GCAFP, F2, S2, and P7 (**G**). Data points represent mean values calculated from two separate experiments.

In order to further confirm the purified effect, the thermal hysteresis activity, the ice contents of GCAFP, F2, S2, and P7 prepared in isolation, and the purification stages with high antifreeze activity were analyzed and compared. The DSC curves of the freezing and melting processes for the GCAFP, F2, S2, P7, and BSA solutions are shown in Figure 4A–E. The calculated thermal hysteresis activities (THA) and ice fractions (φ) at different T_h_, based on the DSC curves, were plotted and are shown in Figure 4F,G, respectively.

As a non-antifreeze standard protein, BSA was usually used as a control. As shown in Figure 4A, the recrystallization of the melted portion started immediately after the temperature dropped, and the exothermic peak appeared without delay, indicating that the BSA solution had no thermal hysteresis activity. However, compared with the DSC curves of BSA, a delayed onset of refreezing temperature (T_o_) was observed for GCAFP, with an increase from −0.8 to −1.4 °C as the T_h_ increased from 1.0 °C to 1.3 °C (Figure 4A). A delayed onset of refreezing temperature (T_o_) was observed for F2, with an increase from 0 to −1.72 °C as the T_h_ increased from 0.80 to 1.0 °C (Figure 4B). A delayed onset of refreezing temperature (T_o_) was observed for S2, with an increase from −1.89 to −5.25 °C as the T_h_ increased from −1.0 to −0.7 °C (Figure 4C). A delayed onset of refreezing temperature (T_o_) was observed for P7, with an increase from −1.22 to −3.99 °C as the T_h_ increased from 0.70 to 1.1 °C (Figure 4D). These results indicate that the GCAFP, F2, S2, and P7 solutions displayed thermal hysteresis properties.

Furthermore, the data shown in Figure 4F indicate that the number of ice nuclei (φ) in the equilibrium sample decreased from 19.43% to 3.40% with a rising T_h_ from 0.70 to 1.1 °C. However, higher values were obtained with a smaller ice fraction in the equilibrium sample. When the ice fraction was less than 10%, the THA values of GCAFP, F2, S2, and P7 increased to 2.7, 2.72, 4.55, and 5.09 °C, respectively. In contrast, the THA value for BSA was only 0.057 °C, lower than those for GCAFP, F2, S2, and P7 (Figure 4G, Table 2). In addition, the THA of the I-SP derived from silkworm was reported to reach as high as 0.94 °C when the amount of ice in the solution was less than 10% [15].

The THA of P7 (5.09 °C) from the *Ctenopharyngodon idella* scales was slightly lower than that of the AFPs from pig skin, which was 5.28 °C [11], but higher than that of the AFPs from the beetle and from Ammopiptanthus mongolicus leaves (0.3 °C, and 0.35 °C, respectively) [29] as well as from silkworm (0.94 °C) [15]. According to research by Cao et al. (2016), THA above 0.6 °C was indicative of hyperactive AFPs [11]. Thus, P7 AFPs from *Ctenopharyngodon idella* scales clearly belong to the hyperactive class. Because of its *Ctenopharyngodon idella* scale origin and antifreeze activity, this new antifreeze peptide was named GCFSC-AFPs.

### 3.3. Primary Structure of the Purified GCFSC-AFPs

**Figure 5:**
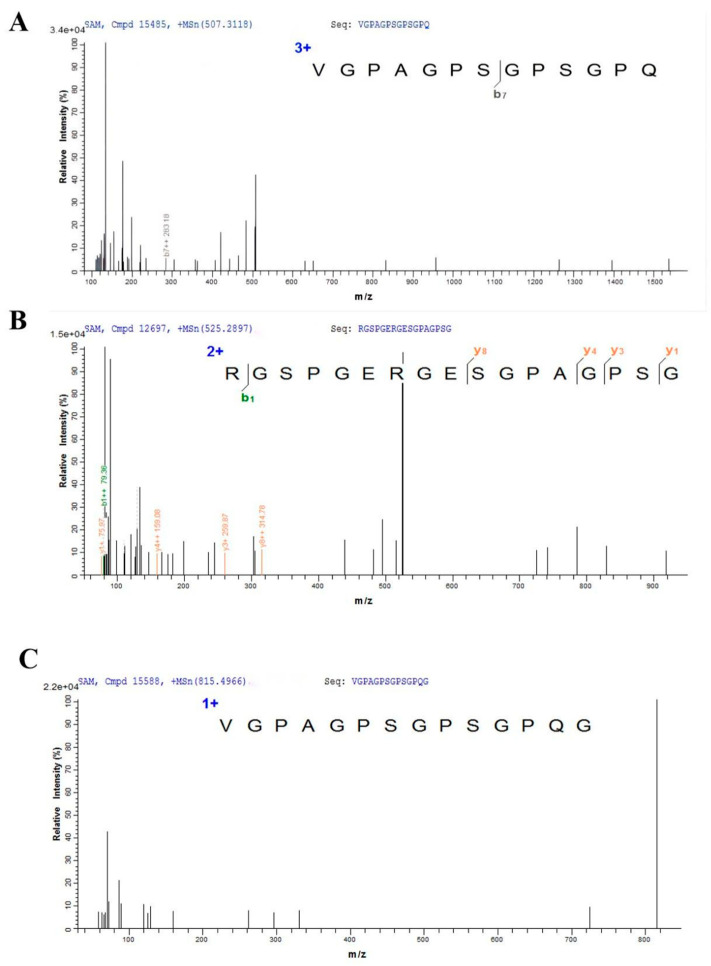
The secondary mass spectrum of GCFSC-AFPs was investigated using Q EXACTIVE LC-MS/MS. As shown in Figure 5, based on electrospray ionization principles, Mascot software, and database scoring, GCFSC-AFPs contained three main peptide amino acids: (**A**): VGPAGPSGPSGPQ, (**B**): RGSPGERGESGPAGPSG, (**C**): VGPAGPSGPSGPQG; the molecular weights of these three peptide of GCFSC-AFPs from the *Ctenopharyngodon* *idella* scales were 1107.54 Da, 1164.32 Da, and 1554.72 Da, respectively.

The peptide sequence of GCFSC-AFPs was investigated by Q EXACTIVE LC-MS/MS. As shown in Figure 5, based on the electrospray ionization principles, Mascot software, and database scoring, GCFSC-AFPs contained three main peptide amino acids: VGPAGPSGPSGPQ, RGSPGERGESGPAGPSG, and VGPAGPSGPSGPQG. The total ion flow diagram of GCFSC-AFPS and database scoring of three peptide sequences are shown in Figure S1 and Table S1 of the Supplementary Materials. The molecular weights of these three peptides of GCFSC-AFPs were 1107.54 Da,1164.32 Da, and 1554.72 Da. Previous studies have found that amino acid composition, molecular mass, peptide sequence, and the ratio of hydrophilic to hydrophobic residues were vital structural characteristics that influenced the antifreeze activity of peptides [2,30]. It was reported that the molecular mass of AFPs with antifreeze activity was usually lower than 3000 Da, and the peptides generally comprised about 10 to 20 residues [6]. Small AFPs are readily adsorbed on the ice crystals [15] and are also more desirable because they can more easily enter cells or food tissues than large proteins or peptides [8].

The peptides sequences of GCFSC-AFPs demonstrated Gly-x1-x2 tripeptide repeats (x1 predominantly proline) with no long-chain aliphatic or aromatic amino acids, which was in good agreement with collagen’s structural properties. AFPs generally have the structural characteristics of a tripeptide repeat sequence (-Gly-Pro-X-, -Gly-X-Pro-, -Gly-X-X- or -T-X-T-) or Pro-Ala-Gly-Tyr as well as being rich in Ala [2]. It has been found that the presence of segment Gly-Pro-x in the collagen peptides was critical for their antifreeze activity. In addition, the contents of Gly, Ala, and Thr are closely related to the antifreeze activity of antifreeze proteins and ice structure proteins [27].

**Figure 6:**
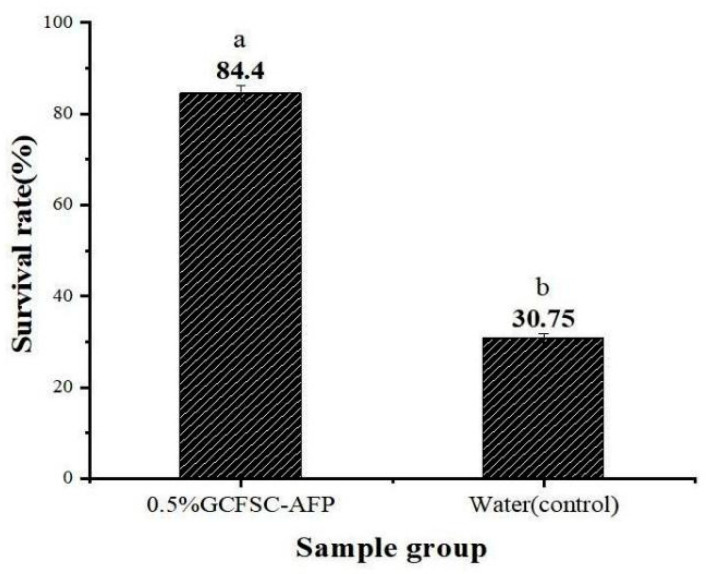
The hypothermia protective effect of 0.5% GCFSC-AFPs on yeast following 1-w of freezing at −20 °C. Data were expressed as the mean ± SD. Note: Means with the same letter in the same column showed no significant difference (*p* ≥ 0.05).

When comparing the three main peptides of GCFSC-AFPs with each other and the antifreeze peptides previously reported, we found that the peptide VGPAGPSGPSGPQ with the molecular weight of 1107.54 Da and the peptide chain VGPAGPSGPSGPQG with the molecular weight of 1164.24 Da had a repetition rate of 92.85% and contained four tripeptide repeats of -Gly-Pro-x. RGSPGERGESGPAGPS with a molecular weight of 1554.61 Da contained 2-Gly-Pro-X and 3-GLy-X-X tripeptide repeats. The THA of GCFSC-AFPs (5.09 °C) was higher than that of other antifreeze polypeptides from fish such as salmon skin (THA0.53 °C), *Scomberomorus niphonius* skin (THA1.7 °C), Tenebrio Molicor (THA3.5 °C).The survival rate of yeast with GCFSC-AFPs reached 84.4% following one week of exposure at −20 ℃ (Figure 6), which was higher than the SaAFP studied by Sun et al. [9].

This may be due to the larger number of repeated sequences of -Gly-Pro-X tripeptide and the higher contents of characteristic amino acids Gly and Pro than the above antifreeze peptides.

### 3.4. Molecular Modeling of GCFSC-AFPs-Ice Interaction

Previous studies revealed that molecular dynamic simulation (MDs) has great advantages in revealing the mechanism of the antifreeze activity of peptides by exploring the process of ice growth [31] and the inhibition of ice crystallization by peptides [32]. In order to demonstrate the mechanism of three antifreeze peptides in inhibiting the crystallization of water molecules, MDs was used to investigate the interaction from the molecular level. Prior to MDs, we first constructed the 3D structure of GCFSC-AFPs based on homology modeling. Then the structure was evaluated through a Ramachandran plot, and the disfavored conformations were optimized by MDs. The appropriate model of GCFSC-AFPs was obtained and used to study the mechanism of antifreeze activity. Subsequently, different peptides were placed in the water phase to observe whether the crystallization process of water molecules was inhibited.

**Figure 7:**
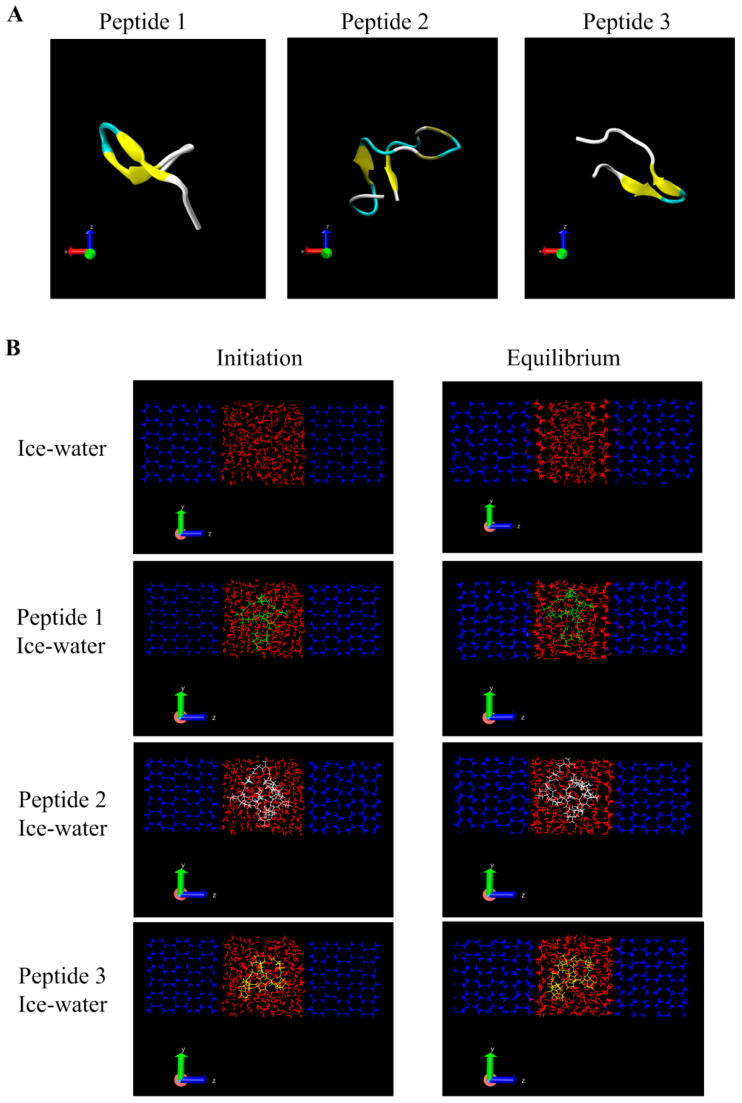
The molecular structure of GCFSC-AFPs (three peptides) shown in a secondary structure model (**A**) and a snapshot of the ice-water system and the three peptides in the ice-water system (**B**). The left panel represents the initial configuration of the system, and the right panel represents the balanced snapshot. Red, blue, green, white, and yellow represent water, ice, peptide 1, peptide 2, and peptide 3, respectively. Data were expressed as the mean ± SD.

**Figure 8:**
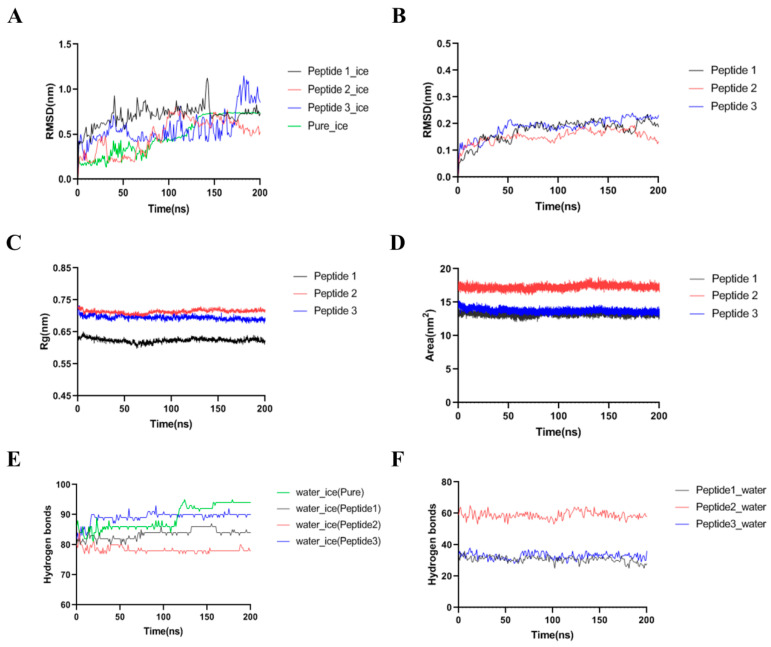
The root mean square deviation (RMSD) of four simulation systems (**A**) and three peptides (**B**). The radius of gyration (**C**) and solvent accessible surface area (**D**) of three peptides during simulation. The numbers of hydrogen bonds formed between water and ice (**E**) and peptides and water (**F**) as a function of time for different systems. Data were expressed as the mean ± SD.

The snapshots are shown in Figure 7. A large number of water molecules could crystallize in the equilibrium state of the ice-water system. However, after adding GCFSC-AFPs, the recrystallization of the water molecules was inhibited. Similarly, Kim et al. found inhibition of ice growth after adding short peptides, especially the peptide enriched in proline residue [32]. The system parameters were further analyzed to evaluate the effect of antifreeze peptides on the growing ice. First, the variations in the root mean square deviations (RMSD) were used to assess the system’s volatility. As shown in Figure 8A, the RMSD values of the ice-water system were below 0.5 Å after 120 ns, indicating the equilibrium of the system. However, the RMSD values of the system exceeded 5 Å after the addition of GCFSC-AFPs, while the RMSD values of GCFSC-AFPs itself were below 0.4 Å (Figure 8B), which meant that the GCFSC-AFPs could affect the crystallization of water. In addition, the structural changes in the GCFSC-AFPs were crucial in maintaining the cryoprotective ability [2,33]. As shown in Figure 8C,D, the radius of gyration and solvent accessible surface area of the three peptides during simulation were counted to evaluate the changes in peptide structure. These values showed small fluctuations throughout the simulation process, indicating that the three peptides could maintain structural stability in the ice-water system, exhibiting excellent cryoprotective ability. Hydrogen bonds also play an essential role in the cryoprotective ability of peptides [15]. As shown in Figure 8E, the number of hydrogen bonds for water and ice was the maximum (93.68 ± 0.78) in the last 50 ns. However, the number of hydrogen bonds formed by water and ice decreased significantly after adding antifreeze peptide 1 (84.38 ± 0.87), antifreeze peptide 2 (78.09 ± 0.61), and antifreeze peptide 3 (89.84 ± 0.51) (*p* < 0.05). Furthermore, the GCFSC-AFPs predominantly formed hydrogen bonds with water rather than ice along with the simulation (Figure 8F). These results indicated that the GCFSC-AFPs interfered with the ice-water interaction and thus inhibited the ice crystallization process. However, Wu, Rong, Wang, Zhou, Wang, and Zhao [15] showed that sericin antifreeze peptides could form hydrogen bonds with ice. This difference might be due to the different systems we used. They used the ice-vacuum system and ignored the effect of the water phase. We therefore concluded that the inhibition of ice crystallization induced by GCFSC-AFPs could be attributed to the interference of the ice-water system and the strong hydrogen bond effect.

## 4. Conclusions

This work documented the preparation and isolation of antifreeze peptides with the ice-water interaction and the subsequent inhibition of the ice crystallization process. 

## Figures and Tables

**Table 1:** The hold and onset temperature (T_h_ and T_o_), recrystallization enthalpy, ice fractions and THA of BSA and *Ctenopharyngodon* *idella* scale hydrolysates after enzymolysis with different papain concentrations, hydrolysis temperatures, pH, and substrate concentrations.

	Papain Concentration (U/g)				Temperature (°C)				pH				Substrate Concentration			
	3000	3500	4000	4500	45	50	55	60	3	4	5	6	1:10	1:15	1:20	BSA
T_h_ (°C)	1.1	1.4	1.3	0.8	0.8	1.2	0.8	1.3	0.7	0.9	1.4	1.1	1.1	0.9	0.8	1.5
T_o_ (°C)	−0.8	−1.26	−1.4	0	0.2	0.1	0.2	0.1	−0.4	−0.4	−1.28	−0.9	−0.9	−0.8	0	1.45
ΔHr (J/g)	−361.1	−401.1	−386.1	−329.8	−404.4	−371	−402.1	−363.7	−249.4	−369.5	−398.9	−409.2	−360.2	−320.9	−347.7	−378.6
φ (%)	0.77	1.67	2.48	1.61	1.10	0.78	0.60	0.08	7.32	2.33	1.48	2.57	3.30	2.30	4.77	0.02
THA (°C)	1.9b	2.66a	2.7a	0.8c	0.60b	1.10a	0.6b	1.2a	1.1c	1.3c	2.68a	2.0b	1.9a	1.7b	0.8c	0.05

Note: Data points represent mean values caculated from two separate experiments. Means with the same letter in the same column showed no significant difference (*p* ≥ 0.05).

**Table 2:** The purification folds of GCFSC-AFPs from the *Ctenopharyngodon idella* scales.

Purification Step	THA (°C)	Purification Fold (Based on Activity)
Hydrolysate GCAFP	2.68bc	1.0b
Sephadex G-50 (Fraction F2)	2.7b	1.03b
SP-Sephadex C-25 (Fraction S2)	4.55ab	1.70a
C18 HPLC (Fraction P7) GCFSC-AFPs	5.09a	1.90a

Note: Means with the same letter in the same column showed no significant difference (*p* ≥ 0.05).

## Data Availability

The datasets generated for this study are available on request to the corresponding author.

## Supplementary Materials

The following supporting information can be downloaded at: https://www.mdpi.com/article/10.3390/foods11131830/s1, Figure S1: Total ion chromatogram of GCFSC-AFPs; Table S1: Peptides table of GCFSC-AFPs by Q Exactive LC-MS/MS.
