# Supplementary material for: The Antifreeze and Cryoprotective Activities of a Novel Antifreeze Peptide from Ctenopharyngodon idella Scales
Source: Foods. 2022 Jun 22;11(13):1830. doi: 10.3390/foods11131830 (PMC9265620; doi:10.3390/foods11131830)
Supplement: Supplementary file 1 [file foods-11-01830-s001.zip › foods-1720136-supplementary.pdf]

Supplementary material

Antifreeze and cryoprotective activities of a novel antifreeze peptide from *Ctenopharyngodon Idella* scales

Meizhu Dang [a,b], Ruifeng Wang [a], Yangyang Jia[a], Jing Du[a,c], Ping Wang [b], Yawei Xu [a], Chunmei Li [a] *

[a]College of food science and technology, Huazhong Agricultural University, Hubei, Wuhan, 430072, China

[b]Henan University of Animal Husbandry and Economy, Henan,Zhengzhou, 450002,China

[c]College of Food Science and Engineering, Wuhan Polytechnic University, Wuhan 430023, China

* Corresponding author: Dr. Chunmei Li

College of Food Science and Technology, Huazhong Agricultural University, Wuhan, Hubei 430070, PR China

Tel and Fax: 87282966; E-mail address: lichmyl@mail.hzau.edu.cn

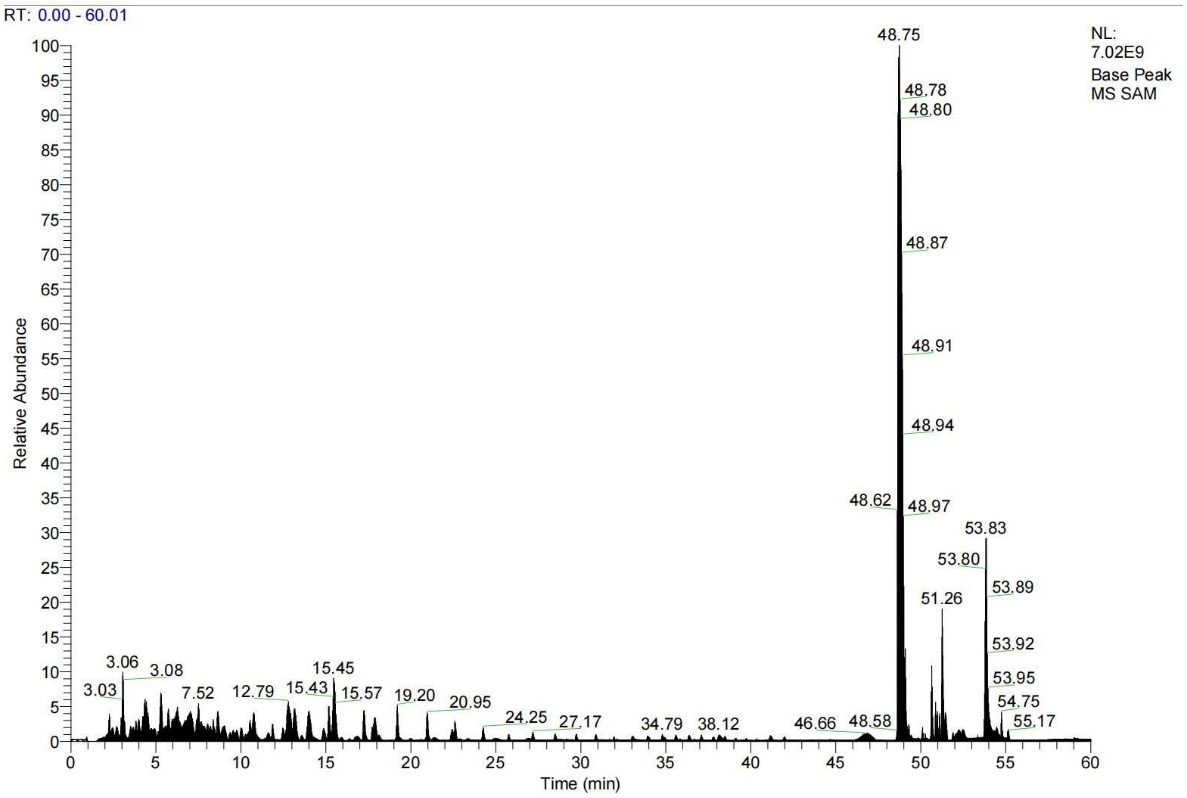

Figure S1. Total ion chromatogram of GCFSC-AFPs

The peptide sequence of GCFSC-AFPs was investigated by Q EXACTIVE LC-MS/MS. The total ion flow diagram of GCFSC-AFPS was obtained, as shown in Fig. S-1. The Raw mass spectrometry test files were retrieved by Uniport database with Mascot software for retrieval and comparison. The selection principle of protein identification results by mass spectrometry is as follows: a score greater than 20 is considered reliable, a score greater than 60 is considered more reliable, and a score greater than 60 is considered to be the most reliable (Zhang Yanjie, 2017). After screening, there were three peptide sequences with scores greater than 60 points: peptide VGPAGPSGPSGPQ scored 74.52, peptide RGSPGERGESGPAGPSG scored 64.29, and peptide VGPAGPSGPSGPQG scored 61.30, as shown in Table S-1. The secondary mass spectrometry of the three peptides is shown in Fig. 5.

Table S1 Peptides table of GCFSC-AFPs by Q Exactive LC-MS/MS

Sequence	Calc.Mass	Reference	Charge	PI	Score
VGPAGPSGPSGPQ	1107.54294	tr|E2IPR2|E2IPR2_CTEID	1	5.5	74.52
RGSPGERGESGPAGPSG	1554.72552	tr|A0A498MWG9|A0A498MWG9_LABRO/tr|A0A498MWD6|A0A498MWD6_LABRO	2	6.14	64.29
VGPAGPSGPSGPQG	1164.56441	tr|E2IPR2|E2IPR2_CTEID	1	5.5	61.3
